# Characterizing the Neuroimaging and Histopathological Correlates of Cerebral Small Vessel Disease in Spontaneously Hypertensive Stroke-Prone Rats

**DOI:** 10.3389/fneur.2021.740298

**Published:** 2021-11-30

**Authors:** Yousef Hannawi, Eder Caceres, Mohamed G. Ewees, Kimerly A. Powell, Anna Bratasz, Jan M. Schwab, Cameron L. Rink, Jay L. Zweier

**Affiliations:** ^1^Division of Cerebrovascular Diseases and Neurocritical Care, Department of Neurology, The Ohio State University, Columbus, OH, United States; ^2^Division of Cardiovascular Medicine, Department of Internal Medicine, Davis Heart and Lung Research Institute, The Ohio State University, Columbus, OH, United States; ^3^Department of Pharmacology and Toxicology, College of Pharmacy, Al-Azhar University, Cairo, Egypt; ^4^Department of Biomedical Informatics, The Ohio State University, Columbus, OH, United States; ^5^Small Animal Imaging Core, Davis Heart and Lung Research Institute, The Ohio State University, Columbus, OH, United States; ^6^Belford Center for Spinal Cord Injury, The Ohio State University, Columbus, OH, United States; ^7^Department of Neurology, The Ohio State University, Columbus, OH, United States; ^8^Department of Physical Medicine and Rehabilitation, The Ohio State University, Columbus, OH, United States; ^9^Department of Neurosciences, The Ohio State University, Columbus, OH, United States; ^10^Department of Neurosurgery, The Ohio State University, Columbus, OH, United States

**Keywords:** cerebral small vessel disease, magnetic resonance imaging, sensorimotor testing, spontaneously hypertensive stroke prone rat, Wistar Kyoto Rat

## Abstract

**Introduction:** Spontaneously hypertensive stroke-prone rats (SHRSP) are used to model clinically relevant aspects of human cerebral small vessel disease (CSVD). To decipher and understand the underlying disease dynamics, assessment of the temporal progression of CSVD histopathological and neuroimaging correlates is essential.

**Materials and Methods:** Eighty age-matched male SHRSP and control Wistar Kyoto rats (WKY) were randomly divided into four groups that were aged until 7, 16, 24 and 32 weeks. Sensorimotor testing was performed weekly. Brain MRI was acquired at each study time point followed by histological analyses of the brain.

**Results:** Compared to WKY controls, the SHRSP showed significantly higher prevalence of small subcortical hyperintensities on T2w imaging that progressed in size and frequency with aging. Volumetric analysis revealed smaller intracranial and white matter volumes on brain MRI in SHRSP compared to age-matched WKY. Diffusion tensor imaging (DTI) showed significantly higher mean diffusivity in the corpus callosum and external capsule in WKY compared to SHRSP. The SHRSP displayed signs of motor restlessness compared to WKY represented by hyperactivity in sensorimotor testing at the beginning of the experiment which decreased with age. Distinct pathological hallmarks of CSVD, such as enlarged perivascular spaces, microbleeds/red blood cell extravasation, hemosiderin deposits, and lipohyalinosis/vascular wall thickening progressively accumulated with age in SHRSP.

**Conclusions:** Four stages of CSVD severity in SHRSP are described at the study time points. In addition, we find that quantitative analyses of brain MRI enable identification of *in vivo* markers of CSVD that can serve as endpoints for interventional testing in therapeutic studies.

## Introduction

Cerebral small vessel disease (CSVD) is a major public health burden resulting in intracerebral hemorrhage, vascular cognitive impairment, and acute ischemic stroke of lacunar type (1, 2). From a pathophysiological perspective, CSVD refers to the pathological processes that affect the brain small vessels in association with aging and hypertension among other cardiovascular risk factors (1, 3). These various pathological processes result in different manifestations on the brain MRI and histology involving dilatation of the perivascular spaces, demyelination, lacunes, and microbleed formation (1, 3). These heterogeneous phenotypes of the disease in humans have resulted in previous challenges in developing experimental disease models representing the full disease spectrum that is seen in humans on histology and brain MRI (4, 5). Hence, several experimental models have been attempted to simulate certain pathological aspects of the disease. These existing experimental models include hypertensive animals, such as the spontaneously hypertensive rats (SHR) and the spontaneously hypertensive stroke-prone rats (SHRSP) with or without salt loading (4, 6). Additional experimental models utilizing these hypertensive animals have added procedures including the induction of hypoperfusion with carotid artery stenosis or occlusion to accelerate the development of CSVD (4, 7). However, across the studies in the literature, aging SHRSP have been most frequently used as a model for human CSVD (4, 8–12).

Spontaneously hypertensive stroke-prone rats is a genetic model for hypertension that was established from a substrain of SHR through selective breeding starting with the 24th generation and fixing a high stroke susceptibility and severe hypertension (13). SHRSPs develop elevated blood pressure early in their course of their lives and they sustain chronic hypertension afterwards throughout their lifespan (4, 13). Previous histopathological studies of their brain have revealed several features of human CSVD including endothelial dysfunction, blood brain barrier breakdown, stasis in the capillaries, extravasation of red blood cells (RBCs), microbleed formation, and demyelination in some instances (8–12, 14). However, several important controversies regarding CSVD in SHRSP remain, limiting their further widespread application. These first include the lack of consistent time points for the appearance of CSVD lesions and their progression (4, 11, 12). Second, the use of salt loading (Japanese modified diet) to accelerate the occurrence of CSVD lesions leading to a state of hypertensive emergency and pathologies consistent with posterior reversible encephalopathy (PRES) rather than CSVD (15). Third, the potential lack of demyelination in one study limits the use of stroke-free SHRSP as a model for white matter hyperintensities (5, 16). Finally, detailed quantitative studies on the neuroimaging correlates of CSVD in SHRSP are still lacking. A recent study of volumetric analysis of brain MRI in SHR has shown atrophy of the corpus callosum pointing toward the need for additional detailed quantitative MRI studies to identify the neuroimaging correlates of CSVD (17).

Therefore, to fulfill these knowledge gaps, we aimed in this work to identify the temporal evolution of histopathological CSVD lesions in SHRSP and their neuroimaging correlates by using visual and quantitative analyses of brain MRI that are acquired at 9.4T along with associated brain histological examination. We also measure the temporal changes of sensorimotor function in these rats. The main goal of this study is to identify consistent disease phenotypes from the brain MRI and histology and their temporal evolution with corresponding time points that can be used as end points for interventional therapeutic studies in the future. Our results suggest that SHRSP, an animal model of marked hypertension, is a relevant model for human CSVD with the development of consistent CSVD lesions with age and associated changes in brain MRI volumetric measures.

## Materials and Methods

All the study procedures were approved by the Institutional Animal Care and Use Committee (IACUC) at the Ohio State University and they were conducted in compliance with the Public Health Service Policy on Humane Care and Use of Laboratory Animals. The experiments have been reported in compliance with ARRIVE guidelines. Equal numbers of male SHRSP and age-matched male Wistar-Kyoto (WKY) rats were obtained from Charles River Laboratories (Wilmington, MA, USA) at 5 weeks of age. Charles River SHRSPs were received from the National Institute of Health (NIH) colony in 2002. The NIH colony originated from the A3 subline that was transferred to the NIH in 1975 at generation F36. All animals were kept under the same physiological conditions at the University Laboratory Animal Resources (ULAR). Animals were housed two per cage under the same 12 h sleep/wake physiological cycle and had unlimited access (*ad libitum*) to regular lab rat chow (Teklad LM-485 mouse/rat serializable diet; 19.1% of protein, 0.3% of sodium, and 0.8% of potassium) that was obtained from Envigo, Inc., with free access to tap water without additional dietary salt. The rats were randomly divided upon their initial arrival in ULAR from Charles River into four experimental groups, each containing equal numbers of rats (10 SHRSP and 10 WKY) at the beginning of the study. Each group was followed until it reached a preassigned time point including 7, 16, 24, and 32 weeks of age. Brain MRI was performed for randomly selected rats followed by euthanasia on the same day upon reaching these time points. All animal groups were monitored weekly for signs of stroke or intracerebral hemorrhage including unilateral weakness, decreased movement, seizures, or significant weight loss. Weight was monitored weekly as well using a standard weighing scale and a weight loss of 10–20%, indicative of potential stroke was recorded. Animals that had >20% weight loss were euthanized according to IACUC regulations.

### Blood Pressure Measurements

Blood pressure was measured in conscious nonsedated rats using tail-cuff plethysmography device (Visitech Systems, Inc., NC, USA) starting at 6 weeks of age and it was repeated weekly in the majority of rats until they reached their study time points. A series of 10 measurements were performed at each session following a period of acclimation for 10 min in the device. The median of the SBP recordings was calculated for each session. SHRSP was considered hypertensive when its SBP consistently and significantly exceeded the upper limit of WKY SBP.

### Sensorimotor Testing

Sensorimotor testing was performed using open field test to monitor for gait disturbances associated with stroke and to assess the progressive sensorimotor changes that are potentially related to the accumulation of CSVD lesions. Sensorimotor testing was started at 6 weeks of age and it was repeated weekly in all rats until they reached their study time points. Animals were placed in the center of a 1-m X 1-m open field and allowed to freely move for 5 min while being recorded overhead using Anymaze video tracking software (Stoelting, v 4.5, Wood Dale, IL). The software calculates multiple metrics related to the sensorimotor performance, such as total distance traveled, average speed, and total mobile time.

### Brain MRI

Brain MRI was acquired under isoflurane general anesthesia using 9.4 tesla (T) system (BioSpec 93/30USR, Bruker, Billerica, MA) at the Ohio State University Small Animal Imaging Core (SAIC). A total of 28 brain MRIs were acquired in the study including 2 MRIs of the 7-week-old group (1 SHRSP and 1 WKY), 10 of the 16-week-old group (6 WKY and 4 SHRSP), 5 of the 24-week-old group (2 WKY and 3 SHRSP), and 11 of the 32-week-old group (7 SHRSP and 4 WKY). Brain MRI was acquired at 7 weeks of age for the primary purpose of optimization of the sequences as we did not anticipate to detect CSVD lesions at this age. The following sequences were acquired: T_1_-weighted rapid acquisition with relaxation enhancement (RARE) (Repetition time (TR): 1,344.5 ms, echo time (TE): 8.3 ms, rare factor (RF): 4, slice thickness 1 mm, Naver: 3, voxel size: 0.117 X 0.117 X 1 mm^3^), T_2_ weighted RARE (TR: 3,524 ms, TE: 36 ms, RF: 8, slice thickness 1 mm, Naver: 2, voxel size: 0.117 X 0.117 X 1 mm^3^), susceptibility weighted imaging (SWI) (TR: 135.1 ms, TE: 2.9 ms, flip angle: 20 degrees, slice thickness: 0.5 mm, Naver: 12, voxel size: 0.075 X 0.075 X 0.5 mm^3^) and echo-planar diffusion tensor imaging (DTI) (TR: 3,800 ms, TE: 23.5 ms, b-value: 670 s/mm^2^), 30 diffusion directions, 8 segments, Δ/δ: 11/5 ms, slice thickness: 1 mm, interslice distance: 1.5 mm Naver: 1, voxel size: 0.234 X 0.234 X 1 mm^3^. Structural sequences (T_1_w, T_2_w, and SWI) were visualized using Image J software package (https://imagej.nih.gov/ij/) (18) for the presence of any brain abnormalities suggestive of CSVD by one investigator (YH). We defined the potential abnormal lesions on T2w imaging as hyperintensities that are visualized within the brain parenchyma. We observed cerebral spinal fluid in the folds between the hippocampus and the corpus callosum in some WKY and SHRSP (19). However, those were not included among the identified CSVD lesions. On SWI, microbleeds were defined as spots of hypointensities within the brain parenchyma that are not part of a vessel. The identified brain MRI abnormalities were ascertained through the review of an additional author who was blinded to the animal age and type (KAP).

Quantitative volumetric analyses of the intracranial, white matter, and the hippocampal volumes were performed by the investigator YH in Image J software package. Intracranial volume was quantified by manually segmenting the brain on T_1_w imaging starting rostrally where the brain can be first seen and ending caudally where the medulla oblongata disappears. The white matter including the corpus callosum and the external capsule were manually segmented on T_2_w imaging starting rostrally where white matter began to be visible on one slice anterior to the genu of the corpus callosum and ended caudally posterior to the splenium of the corpus callosum. The hippocampus and the dentate gyrus were manually segmented on T_2_w imaging as described before (20). The initial rostral slice was defined by the Cornu Ammonis (CA) and dentate gyrus. The segmentation ended caudally by the loss of contrast between the external capsule and subiculum (20). The volume of interest for each of these structures was calculated by adding the area of the segmented region of interest (ROI) on all of these slices and was subsequently multiplied in the slice thickness. In this study, the hippocampal volume was calculated by combining the bilateral hippocampi.

Diffusion tensor imaging was processed by YH using DSI studio software that is publically available at (http://dsi-studio.labsolver.org/) (21). Following visual inspection for motion artifact and eddy current distortion of the raw diffusion sequences, the diffusion tensor was calculated through standard steps in the DSI studio. Subsequently, the fractional anisotropy (FA), mean diffusivity (MD), and color maps were created. The ROI was drawn on each slice to segment the corpus callosum starting from the genu and ending at the level of the splenium. The average FA and MD values were calculated for the segmented corpus callosum. The external capsule was segmented bilaterally at the same levels of the corpus callosum on the color maps starting lateral to the corpus callosum where the external capsule bundle was seen. The average FA and MD of the bilateral external capsules were calculated. These two white matter structures were selected since they were consistently identified in all animals and based on our previous studies showing their impairment in individuals with a history of hypertension (22, 23).

### Euthanasia and Histological Analysis

Euthanasia was performed using CO_2_ inhalation followed by decapitation. Subsequently, the brain was extracted and washed carefully in phosphate-buffered saline (PBS) solution for 2 min. Sections were performed using a standard rat brain matrix and 0.5 cm of the midsagittal brain volume was taken for subsequent fixation in formaldehyde for 48 h. Sections for histological analysis were all performed at the level of the posterior corpus callosum (splenium) and hippocampus. Standard hematoxylin and eosin (H&E) staining was performed from the processed paraffin blocks. Myelin staining was performed using luxol fast blue (LFB) staining. Histological slides were subsequently scanned using the slides of Axio Scanner system (Carl Ziess, Inc. Oberkochen, Germany). The slides were reviewed for the presence of histopathological lesions that are consistent with human CSVD according to well-established definitions (24). The following pathologies were recorded: lacunes defined as cavitating infarcts, arteriosclerosis defined as concentric hyaline thickening of the small arteries, hemosiderin deposits recorded by the presence of hemosiderin on H&E in the brain tissue or perivascular spaces for perivascular hemosiderin, and loss of myelin (demyelination) assessed on LFB staining in the corpus callosum and the external capsule. Extravasation of RBCs outside the vessel lumen was recorded. Enlargement of perivascular in the brain of SHRSP was assessed in comparison to WKY and according to a previously published scale (25). Slides were independently reviewed by YH and the CSVD lesions were ascertained by an independent review by another investigator (JMS) who was blinded to the type and age of the animals.

### Statistical Analysis

Statistical analyses were completed in Stata 9.2 (College Station, TX). Descriptive statistics of continuous data were presented as means and SD. Categorical data were presented as percentages. Group comparisons were completed using *T*-test, Mann–Whitney *U* test, and Fisher Exact test, as appropriate. The *P* < 0.05 was considered statistically significant for all tests. Histogram charts, scatter plots, and trend figures were generated in Microsoft Excel 2016 (Redmond, WA).

## Results

Eighty age-matched male rats (40 WKY and 40 SHRSP) were included in the study. Two SHRSP expired unexpectedly at the beginning of the study and the remaining (78 rats) completed all the study procedures. None of the rats showed signs of stroke or had significant weight loss during the experiment.

### Systolic Blood Pressure Data

**Figure 1:**
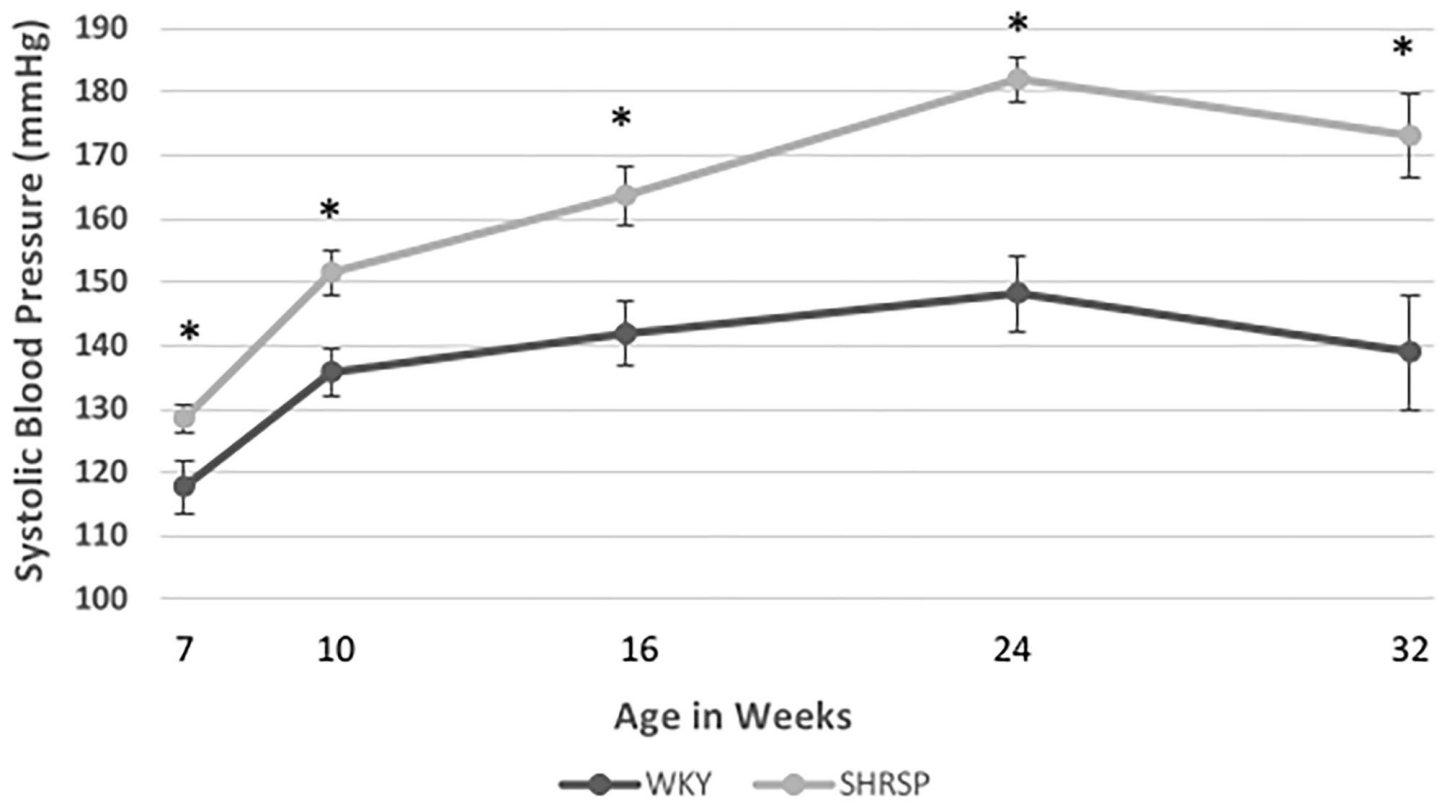
Temporal changes of systolic blood pressure (SBP) in WKY and SHRSP mm Hg (millimeter of mercury) at 7 (21 WKY, 17 SHRSP), 10 (26 WKY, 23 SHRSP), 16 (15 WKY, 15 SHRSP), 24 (8 WKY, 5 SHRSP), and 32 (7 WKY, 5 SHRSP) weeks of age. Throughout the experiment, SHRSP had statistically significant higher SBP compared to WKY. At the beginning of the experiment, SHRSPs were still not hypertensive. Subsequently, SHRSP developed hypertension which was sustained throughout their lifespan. Data are presented as means and standard errors. WKY, Wistar Kyoto Rats; SHRSP, Spontaneously Hypertensive Stroke-Prone Rats (^*^ represents statistical significant difference of *P* < 0.05).

At the beginning of the experiment, SBP of SHRSP was within the normal limits. However, it was significantly higher than WKY (SBP at 7 weeks: SHRSP 128.5 ± 9.4 mmHg vs. WKY 117.8 ± 19.7 mmHg, *P* = 0.035). Subsequently, SHRSP developed elevation in its blood pressure (SBP at 10 weeks: SHRSP 151.5 ± 17 mmHg vs. WKY 135.8 ± 19 mmHg, *P* = 0.004) and sustained a chronic hypertension afterwards throughout the experiment (SBP at 24 weeks: SHRSP 182 ± 6.2 mmHg vs. WKY 148 ± 23 mmHg, *P* = 0.0011). The temporal changes of SBP throughout the study time points are shown in Figure 1 and Supplementary Table 1.

### Sensorimotor Testing

**Figure 2:**
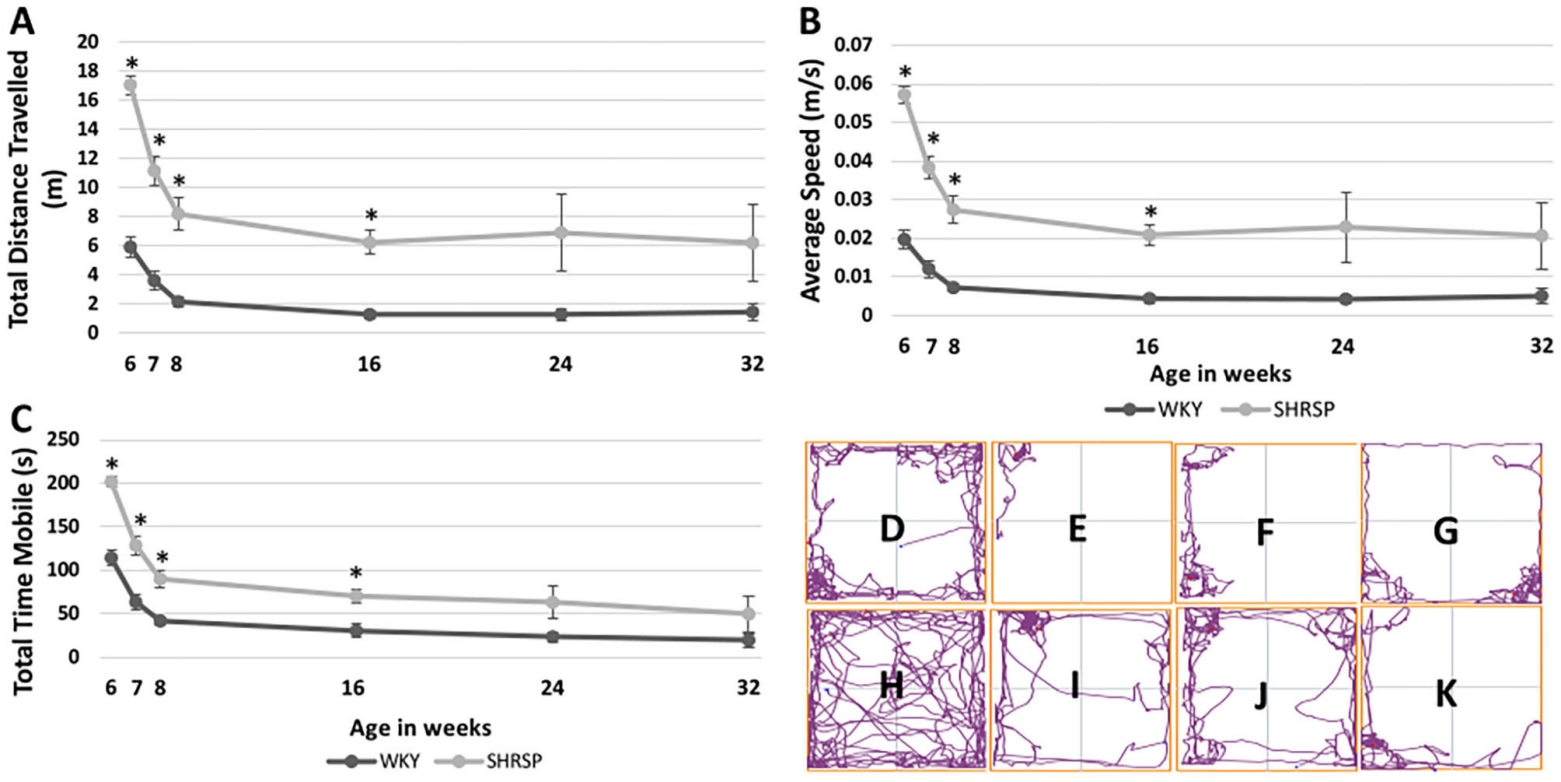
Temporal changes of sensorimotor testing in WKY and SHRSP presented at 6, 7, 8, 16, 24, and 32 weeks of age. Compared to WKY, SHRSP were found to have motor restlessness as represented by a longer total distance traveled during the testing period **(A)**, higher average speed **(B)** and longer total mobile time **(C)**. The differences between SHRSP and WKY, were statistically significant at 6, 7, 8, and 16 weeks of age while they became not statistically different afterwards. Head tracing of one WKY at [6 **(D)**, 16 **(E)**, 24 **(F)**, and 32 **(G)** weeks of age] and one SHRSP at the same time points [6 **(H)**, 16 **(I)**, 24 **(J)**, and 32 **(K)**] weeks of age is shown which visually demonstrates larger decrease in activity during the experiment in SHRSP compared to WKY. WKY, Wistar Kyoto Rats; SHRSP, Spontaneously Hypertensive Stroke-Prone Rats; m: meter; s, second, m/s, meter per second; ^*^, statistically significant difference of *P* < 0.05.

The temporal changes of sensorimotor testing measures at the study time points are shown in Figure 2 and Supplementary Table 2. Throughout the experiment, SHRSP showed signs of motor restlessness and hyperactivity compared to WKY. This was represented by longer total distance traveled during the test, higher average speed, and the total mobile time of the animals. At 6 weeks of age, these differences were statistically significant (total distance traveled: SHRSP 17.1 ± 4 m vs. WKY 5.9 ± 4.6 m, *P* < 0.0001, average speed: SHRSP 0.06 ± 0.013 m/s vs. WKY 0.02 ± 0.016 m/s, *P* < 0.0001, and total mobile time: SHRSP: 202 ±3 4.1 s vs. WKY 114.1 ± 59.3 s, *P* < 0.0001). These metrics subsequently decreased between 6 and 8 weeks of age in SHRSP and WKY due to apparent habituation to the test (Figure 2). The differences in these metrics were no longer statistically significant at 24 weeks of age and they remained not statistically significant until the end of the experiment (at 32 weeks of age: total distance traveled SHRSP 6.2 ± 7.5 vs. WKY1.5 ± 1.9 m, *P* = 0.12, average speed: SHRSP 0.02 ± 0.025 m/s vs. WKY 0.005 ± 0.007 m/s, *P* = 0.12, and total mobile time: SHRSP: 49.7 ± 57 s vs. WKY 19.5 ± 26 s, *P* = 0.2).

### Brain MRI

**Figure 3:**
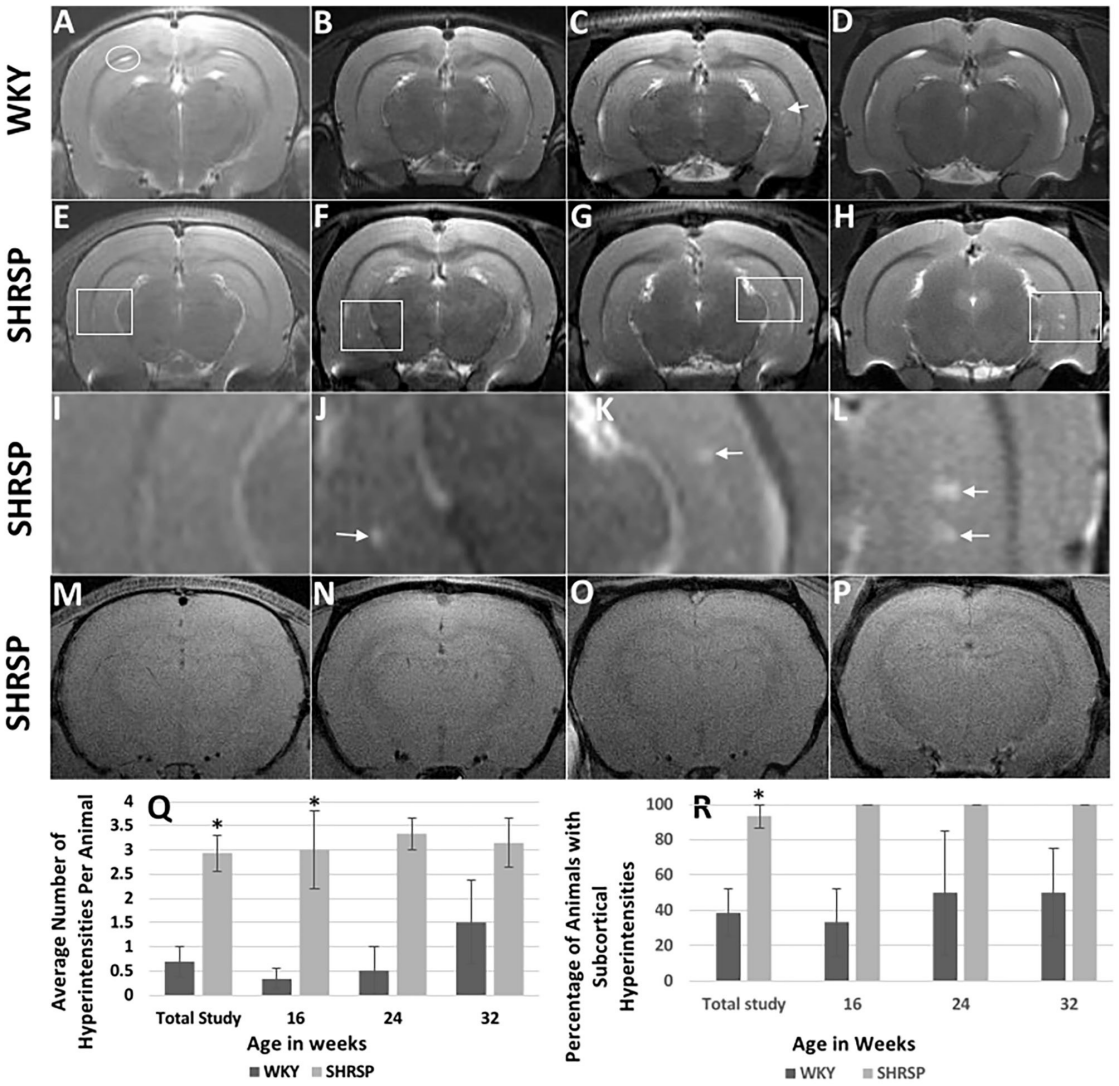
Brain MRI findings in WKY and SHRSP including T2w imaging **(A–L)** and susceptibility weighted imaging (SWI) **(M–P)** at the study time points. Compared to WKY, brain MRI T2w imaging of SHRSP showed progression of subcortical hyperintensities with age in terms of number and size. **(Q)** and **(R)** show the increase in lesion numbers per animal and percentage of animals which had visible lesions. At 7 weeks of age, brain MRI did not show T2w imaging subcortical hyperintensities in WKY **(A)** and SHRSP **(E)**. Early T2w imaging hyperintensities were seen in the hippocampus of SHRSP at 16 weeks **(F)** while they were largely absent in WKY **(B)**. Faint subcortical hyperintensities were visible in some WKY at 24 [arrow in **(C)**] and 32 weeks of age while all SHRSP showed larger and more frequent subcortical hyperintensities at 24 **(G)** and 32 **(H)** weeks of age. White squares in **(E–H)** highlight areas of the hippocampi where subcortical hyperintensities were seen in SHRSP. **(I–L)** show four-time magnification of the areas within the white squares in **(E–H)**. Figure **(I)** demonstrates normal magnified view of the hippocampus at 7 weeks of age. Arrow in J demonstrates small subcortical hyperintensity at 16 weeks of age while arrows in **(K)** and **(L)** demonstrate larger subcortical hyperintensities at 24 and 32 weeks of age reaching a maximum of 0.39 mm in diameter at 32 weeks of age **(L)**. Both WKY and SHRSP showed dilatation of the cerebral spinal fluid folds between the hippocampus and corpus callosum [circle in **(A)**]. Microbleeds were not visible on SWI sequences in SHRSP throughout the experiment at 7 **(M)**, 16 **(N)**, 24 **(O)** and 32 **(P)**, weeks of age. WKY, Wistar Kyoto Rats; SHRSP, Spontaneously Hypertensive Stroke-Prone Rats; ^*^ statistically significant difference of *P* < 0.05. Data represent the mean and standard error of the mean.

None of the brain MRI study of the rats had any evidence of ischemic or hemorrhage strokes. In addition, we did not identify areas of abnormal T1 or T2 signals on the brain MRI in the one WKY and one SHRSP at 7 weeks of age (Figure 3). At 16 weeks of age, however, T_2_w imaging hyperintensities measuring approximately 0.156 mm were seen in the hippocampi in all SHRSP (four out of four rats) and 33% of the WKY (two out six rats) (Figures 3B,F,J). These T2w hyperintensities in the hippocampi progressed with age in size and number in SHRSP while they remained similar in size in WKY at the later study time points (Figures 3C,D,G,H,K,L,Q,R). By 32 weeks of age, T_2_w imaging hyperintensities were seen in the hippocampi of all SHRSP (seven out of seven rats) and 50% of the WKY (two out of four rats) (Figure 3R). These T2w hyperintensities were larger and more frequent in SHRSP compared to WKY. The average number of these T2w imaging hyperintensities was higher at each study time point in SHRSP compared to WKY and in the total study animals (SHRSP: 2.93 ± 1.4 vs. WKY 0.69 ± 1.1, *P* = 0.0005) (Figure 3Q, Supplementary Table 3). Furthermore, the lesion size reached a maximum of 0.391 mm in SHRSP at 32 weeks and it remained about the same size of 0.16 mm in WKY. Finally, the percentage of animals in our study with any subcortical hyperintensities was also significantly higher in SHRSP in comparison to WKY (SHRSP 93.3%, 95% CI: (68.1–99.8) vs. WKY 38.5%, 95% CI: (13.9–68.4), *P* = 0.004). Outside the hippocampi, we did not observe abnormal T_2_w signal hyperintensities in the white or gray matter in WKY or SHRSP. Visual examination of SWI failed to detect the sufficient increase of susceptibility in the brain parenchyma that is typically seen in microbleeds of humans in any of the study, either WKY or SHRSP (Figures 3M–P). T2 signal hyperintensity consistent with CSF in the folds between hippocampus and corpus callosum were seen in WKY and SHRSP (Figure 3A).

**Figure 4:**
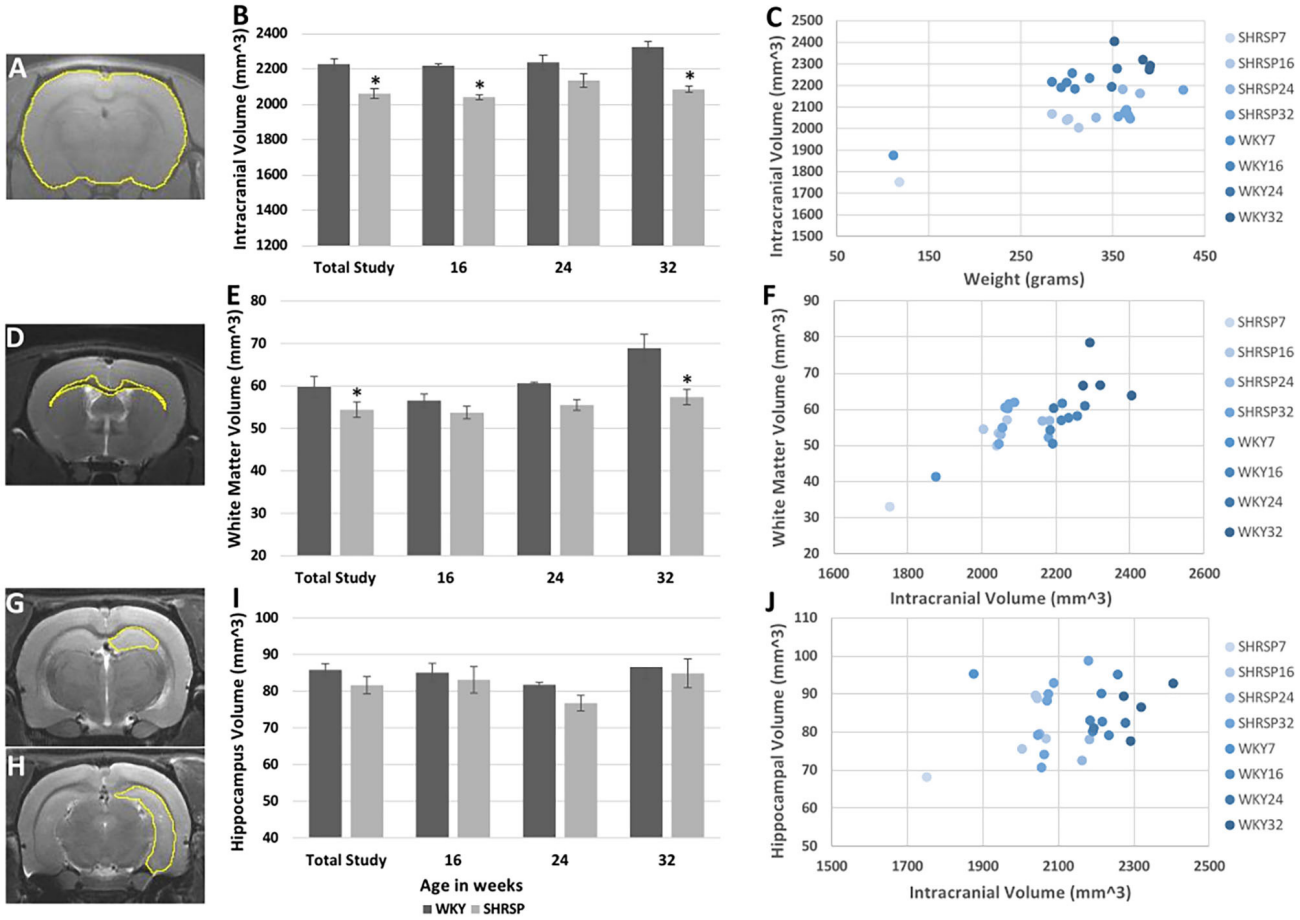
Volumetric analyses of the intracranial **(A–C)**, white matter **(D–F)**, and hippocampal **(G–J)** volumes in WKY and SHRSP show smaller volumes in SHRSP compared to WKY. Manual segmentation of one slice is shown on T1 sequence for the intracranial content **(A)**, T2w imaging for the white matter **(D)**, and hippocampus **(G,H)**. The intracranial volume was statistically and significantly smaller in SHRSP compared to WKY in the total study and at the study time points **(B)**. White matter volume was statistically and significantly smaller in SHRSP in both the total cohort and at 32 weeks **(E)**. Finally, while the hippocampal volume was smaller in SHRSP, the difference was not statistically significant **(I)**. Scatter plot presentation of the data is presented in **(C)**, **(F)**, and **(J)** in which SHRSP and WKY at each time point is presented with a distinct color. Figure **(C)** shows the difference between SHRSP and WKY using intracranial volume (vertical axis) and weight (horizontal axis). A lesser difference is seen using white matter volume (vertical axis) with intracranial volume on the horizontal axis **(F)**. Finally, animals do not appear to be different according to the hippocampal volume (vertical axis) **(J)**. WKY, Wistar Kyoto Rats; SHRSP, Spontaneously Hypertensive Stroke-Prone Rats; ^*^ Statistically significant difference of *P* < 0.05. Data in **(B)**, **(E)**, and **(I)** represent the mean and standard error of the mean.

Volumetric analyses showed statistically significant smaller intracranial volume in SHRSP compared to WKY (Figures 4A–C, Supplementary Table 4) (intracranial volume in the total study rats: SHRSP 2,059 ± 100.1 mm^3^ vs. WKY 2,226 ± 121.7 mm^3^, *P* = 0.0001). This difference was seen across all study time points and it became larger at 32 weeks of age. We observed variability in the intracranial volume among the study groups in both WKY and SHRSP. While, the intracranial volume was smaller in SHRSP at 32 weeks compared to SHRSP at 24 weeks, this difference was not statistically significant. Similarly, white matter volume was significantly smaller in SHRSP compared to WKY (Figures 4D–F) (total animals in the study: SHRSP 54.4 ± 7.1 mm^3^ vs. WKY 59.8 ± 8.8 mm^3^, *P* = 0.045). We also observed the largest difference in the white matter volume between WKY and SHRSP at 32 weeks of age. However, we did not identify significant reduction of the white matter volume with age in WKY or SHRSP. The hippocampal volume was smaller in SHRSP compared to WKY. However, this difference was not statistically significant (Figures 4G–J) (total animals in the study: SHRSP 81.6 ± 9.1 mm^3^ vs. WKY 85.8 ± 6.1 mm^3^, *P* = 0.12). Scatter plot analyses of the intracranial, white matter, and hippocampal volumes are presented in Figures 4C,F,J. The scatter plot graphically reflected the differences among the groups showing larger differences in ICV followed by the white matter volume between SHRSP and WKY at each study time point.

**Figure 5:**
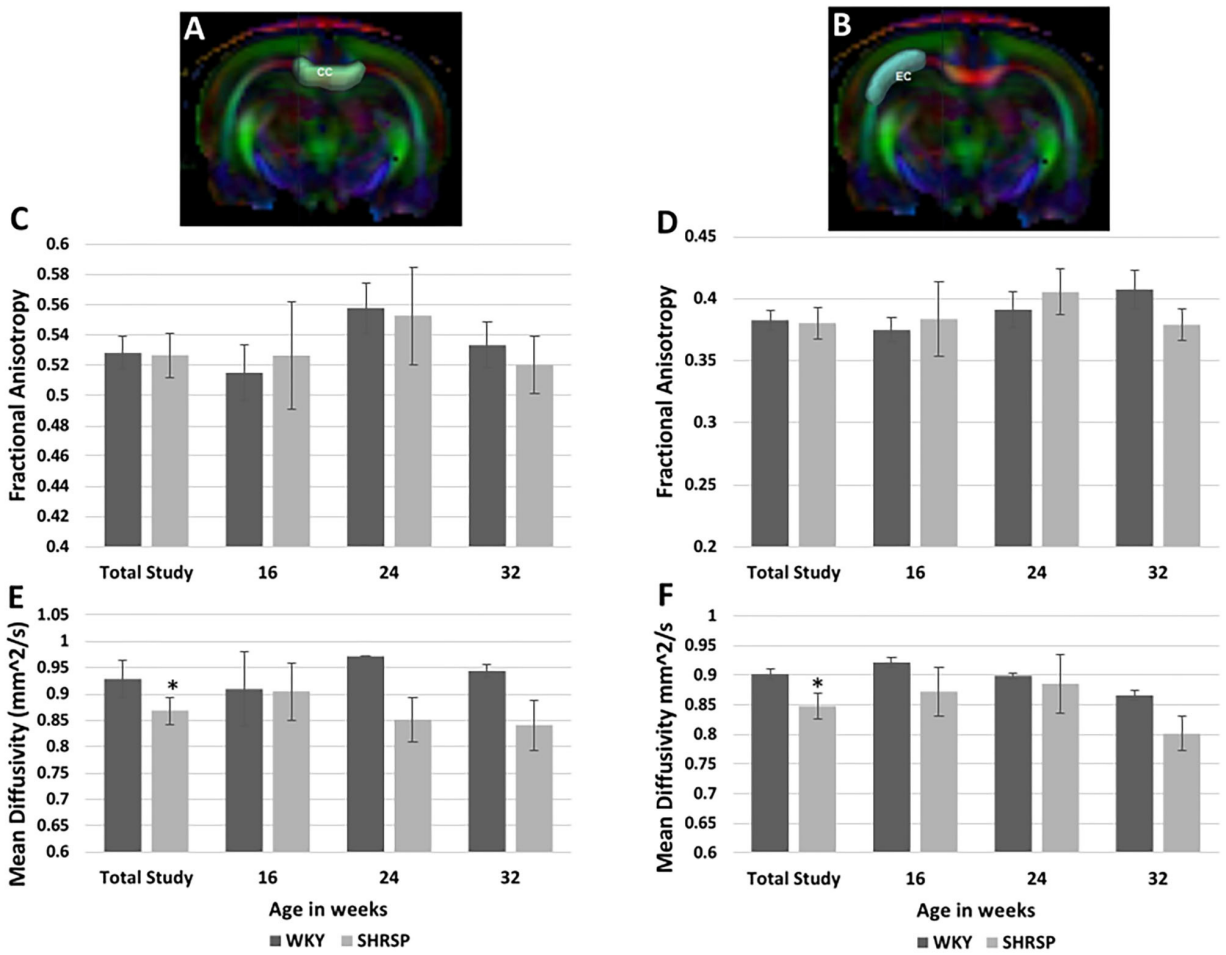
Diffusion Tensor Imaging (DTI) analyses of the corpus callosum and external capsule in SHRSP and WKY show higher mean diffusivity (MD) values in WKY compared to SHRSP in both structures **(E,F)**. This difference was only statistically significant in the total study animals. The fractional anisotropy (FA) was not statistically different between WKY and SHRSP in both structures **(C,D)**. Segmentation of corpus callosum is shown on one slice of the color map in **(A)** (CC) and the external capsule segmentation is shown at the same level in **(B)** (EC). CC, corpus callosum; EC, external capsule; mm^2^/s: square milli*meter per second*; ^*^ statistically significant difference of *P* < 0.05. Data represent the mean and standard error of the mean.

Diffusion Tensor Imaging analysis (Figures 5A,B) showed no difference in the fractional anisotropy (FA) of the corpus callosum or the external capsule between SHRSP and WKY across the study time points (total study animals: FA Corpus callosum: WKY 0.528 ± 0.037 vs. SHRSP 0.527 ± 0.052, *P* = 1, FA External capsule: WKY 0.383 ± 0.029 vs. SHRSP 0.38 ± 0.045, *P* = 0.87) (Figures 5C,D, Supplementary Table 4). However, both the corpus callosum and the external capsule exhibited significantly higher mean diffusivity (MD) in WKY compared to SHRSP in the total cohort (MD Corpus callosum WKY 0.928 ± 0.119 mm^2^/s vs. SHRSP 0.869 ± 0.093, *P* = 0.009, MD external capsule WKY 0.902 ± 0.028 mm^2^/s vs. SHRSP 0.8480 ± .076 mm^2^/s, *P* = 0.03). Similarly, MD was higher in WKY vs. SHRSP at each time point. But it did not reach statistical significance (Supplementary Table 4, Figures 5E,F).

### Histological Findings

**Figure 6:**
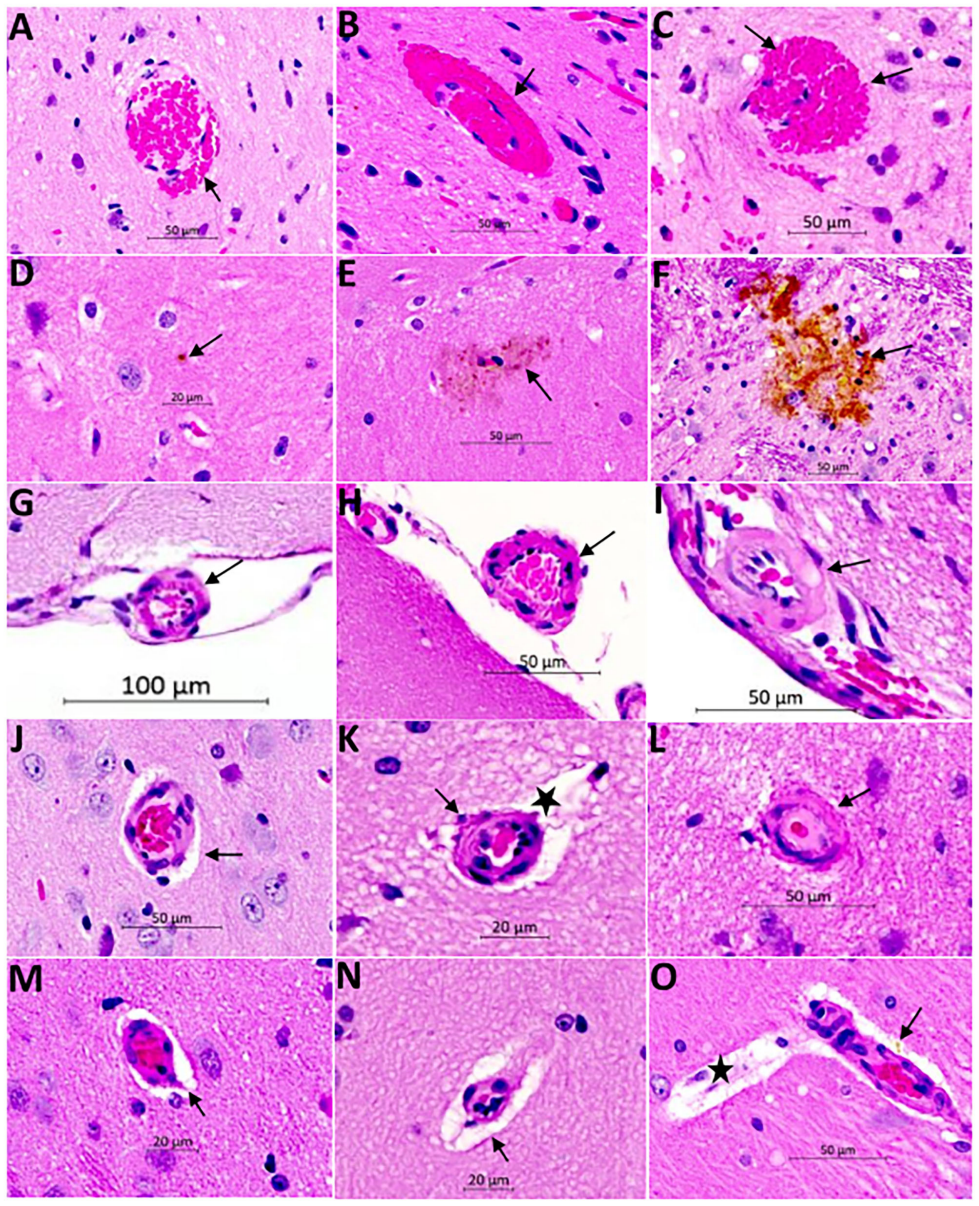
Hematoxylin and Eosin (H&E) staining of the brain of SHRSP demonstrates CSVD lesions including extravasation of red blood cells and microbleed formation **(A–C)**, hemosiderin deposition **(D–F)**, thickening and hyalinization of vessel wall **(G–L)**, and enlargement of the perivascular spaces **(M–O)**. Sections were obtained from the mid sagittal brain area at the level of the dorsal and ventral hippocampus and images were captured at (20X) magnification. Early red blood cells extravasation is encountered consistently at 16 weeks of age [arrow in **(A)**] which increases in size with age at 24 weeks [arrow in **(B)**] and 32 weeks [arrows in **(C)**]. Hemosiderin disposition is seen later in life at 24 weeks [arrows in **(D)** and **(E)**] and it increases in size at 32 weeks of age [arrow in **(F)**]. The small and medium size vessels in the subarachnoid space undergo structural changes showing wall thickening in SHRSP. These vessels appear normal at 7 weeks of age [arrow in **(G)**] while they develop mild thickening at 16 weeks of age [arrow in **(H)**] and lipohyalinosis at 24 weeks of age [arrow in **(I)**]. The penetrating small vessels undergo similar changes as well with age **(J–L)**. They largely appear normal at 7 weeks of age **(J)**, while mild thickening is seen at 16 weeks of age [arrow in **(K)**] and lipohyalinosis is seen at 24 weeks of age [arrow in **(L)**]. Enlargement of the perivascular spaces can be seen as early as 7 weeks [arrows in **(J,M)**] and they increase in size at 16 weeks [star in **(K)** and arrow in **(N)**] and 24 weeks [star in **(O)**]. Capillaries are often congested **(M)**. Perivascular hemosiderin is another histopathological finding starting at 24 weeks [arrow in **(O)**].

**Figure 7:**
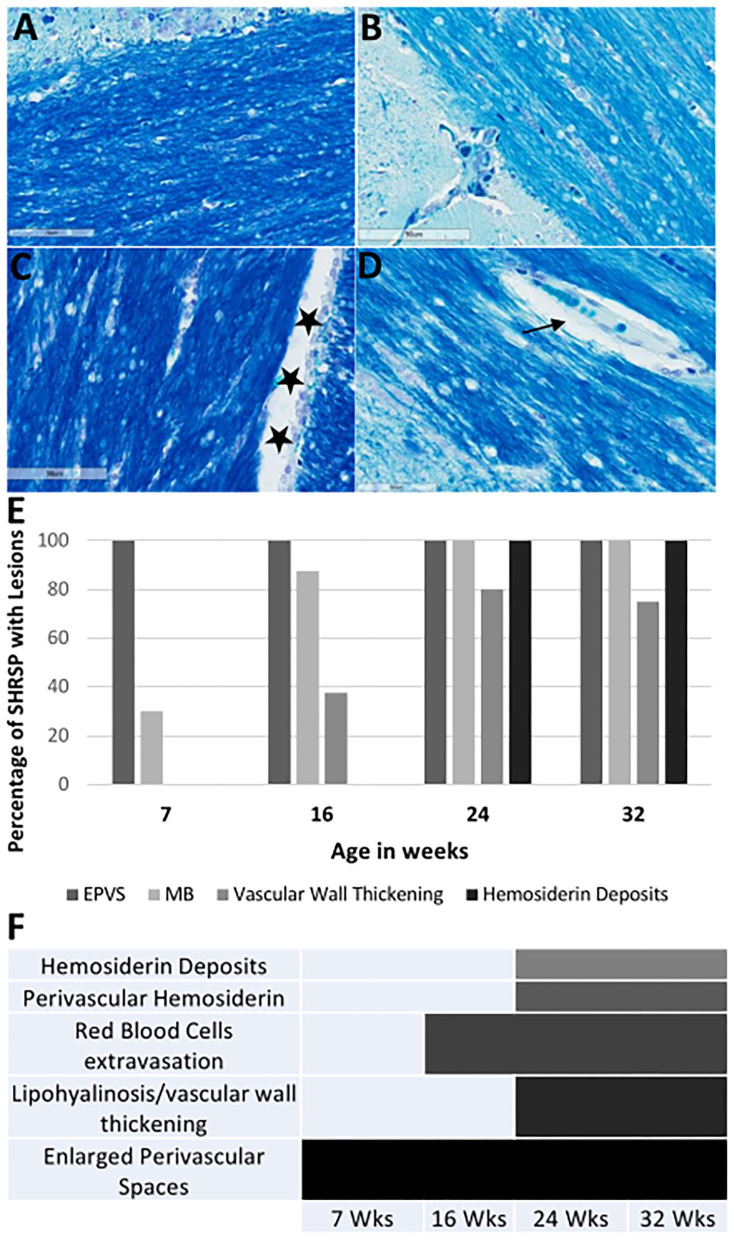
Luxol fast blue (LFB) staining of the corpus callosum and the external capsule in WKY and SHRSP **(A–D)** at 40X magnification. Staining is negative for areas of demyelination. Normal view of the white matter is shown in WKY **(A)** and SHRSP **(B)** at 7 weeks of age. Stars in **(C)** shows an example of cerebral spinal fluid space dilation in a 32-week old WKY. We identified decrease in myelin in SHRSP surrounding the capillaries due to the expansion of the perivascular space [arrow in **(D)**]. The percentage of SHRSP at study time points with each of CSVD lesions are shown in **(E)**. **(F)** Demonstrates the progression of these lesions across age. These results demonstrate that EPVS. are early findings while hemosiderin deposition occur later in life. SHRSP, spontaneously hypertensive stroke-prone rats; EPVS, enlarged perivascular spaces; MB, microbleed; Wks, weeks.

The histopathological findings of CSVD lesions in SHRSP and their temporal progressions across the study time points are shown in Figures 6 and 7. SHRSP showed consistent evolution of histopathological features resembling human CSVD across the study time points including enlarged perivascular spaces, extravasation of RBCs from the capillaries and microbleed formation, hemosiderin deposition and vessel wall thickening, and lipohyalinosis. At 7-weeks of age, the most consistent histopathological findings in SHRSP were mildly enlarged perivascular spaces (Figure 6M) that was seen in all SHRSPs (Figures 7E,F). We identified a microvascular disease associated with the extravasation of RBCs in three animals (30%) at 7 weeks of age (Figure 7E). At 16 weeks of age, SHRSP showed consistent microvascular disease associated with small extravasation of RBCs from the capillaries and capillary congestion (Figure 6). In addition, the enlarged perivascular spaces became more prominent (Figures 6K,N). At 24 weeks of age, SHRSP exhibited consistent and prominent CSVD lesions including extravasation of RBCs, capillary congestion/stasis, enlarged perivascular spaces, microbleed formation, and small hemosiderin deposits (Figure 6). Additionally, the majority of animals showed histological findings of lipohyalinosis and vessel wall thickening in both the perforating vessels and subarachnoid medium and small vessels. The hemosiderin deposits became larger at 32 weeks in addition to the presence of the other CSVD lesions. Those hemosiderin deposits were not localized to particular brain regions and they were seen in the cortex and white matter. The LFB staining did not identify significant areas of demyelination in WKY and SHRSP (Figure 7). However, a few WKY and SHRSP showed dilatation of the CSF spaces consistent with the brain MRI findings (Figure 7). We did not find histological evidence of lacunes, infarcts, or large intracerebral hemorrhage in any of the animals studied. In correlation with the T2w imaging hyperintensity lesions of the hippocampi, the histological findings of the hippocampi showed areas of enlarged perivascular spaces, capillary congestion, and extravasation of RBCs suggesting that these CSVD lesions are the source of the abnormal MRI signal.

## Discussion

**Figure 8:**
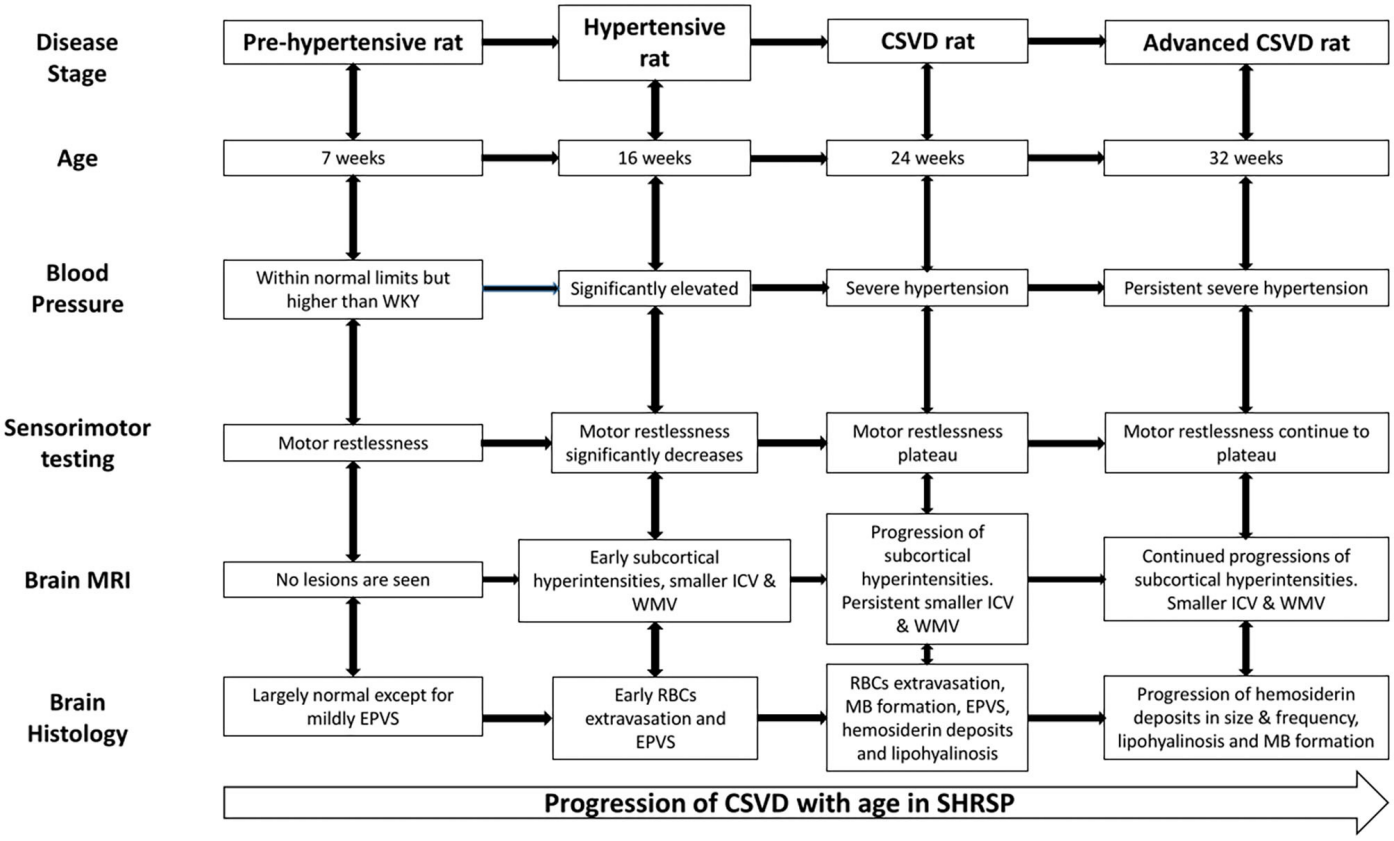
Cerebral small vessel disease (CSVD) stages in SHRSP across age. The pre-hypertensive rat does not show consistent evidence of CSVD except for mildly dilated perivascular spaces but they are restless during the sensorimotor testing. Subsequently, SHRSP develop significant hypertension and they show decrease in restlessness associated with the development of radiographic and histological features of CSVD. The figure details the CSVD correlates at each study time point from the hypertensive rat stage at 16 weeks until the advanced CSVD stage at 32 weeks of age. CSVD, cerebral small vessel disease; EPVS, enlarged perivascular spaces; ICV, intracranial volume; MB, microbleed; RBCs, red blood cell; SHRSP, spontaneously hypertensive stroke-prone rats; WKY, Wistar Kyoto rat; WMV, white matter volume.

Spontaneously hypertensive stroke-prone rat is an animal model for essential hypertension that has been used to study the underlying pathophysiological mechanisms of human CSVD (10–12, 26). In particular, the critical roles of endothelial dysfunction and blood brain barrier impairment in the disease progression have been revealed through these studies (10, 26). However, several questions concerning the histopathological and neuroimaging correlates of CSVD in SHRSP and their progression limiting the use of SHRSP in further therapeutic interventional studies due to the lack of defined disease endpoints remain unanswered (4, 27, 28). Therefore, we aimed in this study to define the neuroimaging and histopathological features of CSVD and their progression at several time points of SHRSP life by utilizing quantitative neuroimaging in association with histopathological analyses. In our results, we demonstrate an age-dependent progression of histopathological findings consistent with CSVD in SHRSP. In correlation, we identified progressive T2w imaging subcortical hyperintensities on the brain MRI with associated smaller intracranial and white matter volumes compared to control WKY. In particular, we characterize four stages of the disease in SHRSP at the study time points (Figure 8). In the pre-hypertensive rats, at 7 weeks of age, the brain MRI and histology are largely negative for CSVD lesions except for mild enlargement of the perivascular spaces. In the hypertensive rat, at 16 weeks of age, there is an appearance of early microvascular disease with enlargement of the perivascular spaces on histology. In correlation, the brain MRI shows subcortical T2w imaging hyperintensities. These findings continue to progress in the CSVD rat stage at 24 weeks of age and advanced CSVD stage at 32 weeks of age in terms of lesion frequency and sizes (Figure 8). In correlation, SHRSP shows a decline in motor restlessness and activity in the open field test across the study time points. Taken together, our findings confirm that SHRSP is a relevant animal model for human CSVD with measurable brain MRI and histological CSVD findings that progress in an age-dependent manner.

Several studies have previously reported brain MRI findings in SHRSP without associated surgical procedures (Table 1) (12, 15, 16, 29–35). The majority of these previous studies have utilized high salt diet administration to accelerate the occurrence of hypertensive disease in SHRSP reporting the findings of cerebral edema or posterior reversible encephalopathy (PRES) that are consistent with a state of hypertensive emergency (15, 29, 30, 32, 34, 35). When regular diet alone was administered, previous studies have reported histological findings of microvascular disease, capillary stasis, and microbleed formation that share similar characteristics with human CSVD (12, 33). When brain MRI was performed in association with a limited number of these studies, white matter hyperintensities or microbleeds were not detected (16, 33). In another study, spontaneous strokes were detected on brain MRI in SHRSP which were fed regular diet when animals were allowed to reach an approximate advanced age of 1.2 years (31). These studies were largely descriptive as they did not employ volumetric analysis of the brain MRI sequences; they utilized lower magnetic field strength ranging between 3.0 and 7.0 T (Table 1) and they did not capture the full life span of SHRSP (36). Our current work extends the previous studies by utilizing a higher magnetic field strength (9.4 T), which allows for higher sensitivity and image resolution than possible at lower fields as well as performing detailed volumetric analyses of the brain, white matter, and hippocampus volumes and assessing the white matter microstructure using DTI. Our findings identified small subcortical T2 hyperintensities that progressed throughout the life of SHRSP and smaller brain and white matter volumes in SHRSP compared to WKY at each of the study time points. Similar findings of smaller brain and white matter volumes were identified as well in SHR compared to WKY using voxel-based volumetric analysis (17). The results of quantitative neuroimaging are in line with previous *ex vivo* volumetric brain studies of SHR showing progressive atrophy and smaller brain volumes (37). This latter study, however, did not assess the microstructural integrity of the brain and white matter in SHR. Since our study design focused on performing neuroimaging and histological analyses at the same time points, we did not perform serial neuroimaging measurements of the same animals. Hence, we are not able to ascertain the magnitude of potential brain and white matter atrophy across age. While, we did not observe any significant decrease in the intracranial and white matter volumes in the WKY and SHRSP across age groups, we found, larger differences between WKY and SHRSP in the intracranial and white matter volumes at 32 weeks of age. Furthermore, 32-week-old SHRSP showed smaller brain volumes compared to that in the 24-week-old SHRSP rats. The increase in the intracranial volume that was seen between 7-week rats and the subsequent time point is due to the growth of animals as previous study showed animal brains continue to maturate within the first 2 months (38). We also believe that variability among animal strains of WKY and SHRSP could have played a role in these findings. On visual examination of the MRI sequences, we identified subcortical T_2_w imaging hyperintensities that were more frequent, larger in size, and appeared at earlier age in SHRSP compared to WKY. These T_2_ hyperintensities were seen in the hippocampus. Based on the associated histological examination of the hippocampus, these areas showed congestion of the capillaries, enlarged perivascular spaces and leakage of RBCs across the blood brain barrier. In addition, as it has been shown before, we identified additional T_2_w signal consistent with the dilatation of the cerebrospinal fluid spaces between the hippocampus and corpus callosum that were seen in WKY and SHRSP. These findings have been attributed before in Wistar rats to the impairment of cerebrospinal fluid transport in these animals (19).

**Table 1:** Previous literature assessing cerebral small vessel disease findings on brain MRI in SHRSP.

**Study and (reference number)**	**Animal strain and (age)**	**Diet**	**MRI field strength and acquisition sequences**	**Analytical approach**	**Findings**
Blezer ELA et al. (29)	SHRSP (8 weeks)	High salt diet (1% NaCl in water)	4.7T, T2W images	Pixel mean intensity to calculate cerebral edema	SHRSP developed cerebral edema in 30 days of high salt diet
Blezer ELA et al. (30)	SHRSP (8 weeks)	High salt diet (1% NaCl in water)	4.7T, T2W images	Analysis of increased T2 relaxation time	Data were used in previous experiment. Study developed objective method for cerebral edema quantification in SHRSP
Brittain JF et al. (16)	WKY & SHRSP (10 months)	Regular diet	7T, T2W image, DTI and GRE	Visual inspection	T2W imaging was negative for WMH and GRE was negative for bleeding. DTI showed increased in FA & modest reduction in MD in corpus callosum in SHRSP compared to WKY
Henning EC et al. (31)	SHRSP (6 months)	Regular diet	7.0T, T1W with/without contrast, T2W, DWI, GRE, ASL.	Visual inspection and quantitative analysis	83% of SHRSP had ischemic lesions on brain MRI. The average age for symptom onset was 1.2 years
Herrison F et al. (15)	SHRSP and SHR (12 weeks).	Regular or high salt diet and 1% NaCl in water	4.7T, T1W, T2W, DWI, ADC, CBF maps.	Visual inspection and CBF analysis	MRI showed T2 hyperintense lesions suggestive of vasogenic edema.
Lee JM et al. (32)	WKY and SHRSP (12 weeks)	High salt diet	3T, T1W with & without contrast, T2W and GRE	Quantitative maps for permeability and visual inspection	SHRSP developed increased vascular permeability prior to development of intracerebral hemorrhage
Mencl S et al. (33)	WKY & SHRSP (26–44 weeks)	Regular diet	3.0T, T1W, T2W, FLAIR, GRE images	Visual inspection	MRI did not detect small perivascular bleeding. But, it detected macrobleed
Schreiber S et al. (12)	WKY and SHRSP (12–42 week)	Regular diet	4.7T, T2W imaging and MR angiography	Visual inspection	2/15 SHRSP had T2 hypeintensitive lesions at 32 weeks of age
Sironi L et al. (34)	WKY, SHR & SHRSP (6 weeks)	High salt diet and 1% NaCl in drinking water	4.7T, T2W imaging and contrast enhanced T1W imaging	Visual inspection and quantitative measurement of blood brain barrier breakdown	SHRSP developed blood brain barrier breakdown and edema
Takahashi M et al. (35)	SHRSP	Funabashi diet and 1% NaCl in drinking water	4.7T, T2W and DWI	Visual inspection and quantitative analysis of ADC maps	Tissue ADC is useful for determination of chronic brain lesions (gliosis vs. edema vs. control vs. cyst)

*ADC, apparent diffusion coefficient; ASL, arterial spin labeling; CBF, cerebral blood flow; DTI, diffusion tensor imaging; DWI, diffusion weighted imaging; FA, fractional anisotropy; FLAIR, fluid-attenuated inversion recovery; GRE, gradient echo; MD, mean diffusivity; NaCl, sodium chloride; SHR, spontaneously hypertensive rat; SHRSP, spontaneously hypertensive stroke-prone rats; T, Tesla; T1W, T1 weighted -; T2W, T2 weighted; WKY, Wistar Kyoto rat; WMH, white matter hyperintensity*.

Despite the presence of microbleeds associated with the extravasation of RBCs and hemosiderin deposits on the histopathological examination, the review of SWI did not reveal susceptibility artifact suggestive of microbleed formation. SWI relies on phase imaging to enhance MRI contrast. This enhancement enables the detection of paramagnetic content in the tissues, such as iron from microbleeds in humans with CSVD (3, 39, 40). There are potentially several reasons for the lack of visualization of microbleeds on SWI in our model. First, a previous report of imaging of larger bleeds on SWI related to traumatic brain injury suggested that microbleeds may temporarily become less visible or invisible in rats (41). Second, the size of the microbleeds that are identified in our study (100–200) μm on histopathology would likely to be too small to detect even at 9.4T, while perhaps at even higher magnetic field strength or with much longer imaging, these might be detected in the future. In addition, the rapid development of intravenous contrast materials associated with SWI acquisition that enables mapping of the microvasculature in these animals at the smaller vascular level promise to provide additional tools to examine the microvasculature in this model (42). This could potentially provide a new approach to identify vascular leak across the BBB.

Using DTI, we identified higher MD values in the corpus callosum and external capsule in WKY compared to SHRSP, while the FA values were not statistically and significantly different. DTI uses the physical properties of water diffusion to map the microstructure of the underlying tissues (43). In the brain healthy white matter, cellular membranes with some contributions from myelination and the packing of axons, skew the directionality of water diffusion, making it less uniform i.e., “anisotropic” (44). This results in the reduction of the FA and increase in the MD values in the abnormal white matter (44). In human, the typical finding of DTI in patients with radiographic evidence of CSVD consists of higher MD and lower FA values compared to those without CSVD which suggest the disruption of the white matter tract integrity at the microstructural level (22, 23, 45). Interestingly, our findings are in line with a previous study that performed DTI of the corpus callosum in SHRSP compared to WKY (16). This phenomenon was previously attributed to the rapid regeneration of white matter in rats compared to humans that could be induced by hypoperfusion (46). Another potential explanation is the dilation of CSF spaces that is seen in Wistar rats that was found to be associated with worse DTI metrics in a previous study (19). These findings argue that DTI may not potentially serve as a good tool for detecting the phenotypes of CSVD in SHRSP.

The histopathological examination of the brain in SHRSP has identified several CSVD lesion types that accumulated with age. The dominant features include microvascular disease, capillary congestion, and extravasation of RBCs. Enlarged perivascular spaces were seen early in life starting at 7 weeks of age, while hemosiderin deposition was a late finding starting at 24 weeks of age. Additional histopathological findings include vessel wall thickening and lipohyalinosis of the medium and small vessels including those vessels on the brain surface and intraparanchymal penetrating vessels. We also identified perivascular hemosiderin deposits in the older animals. Our histopathological findings are consistent with the majority of previous studies of SHRSP describing CSVD histological phenotypes (11, 12). They are also similar to CSVD lesions that are seen in humans except for lack of demyelination and lacune formation (25, 47). This lack of demyelination has suggested that SHRSP may not be a suitable model for white matter hyperintensities in one study (16). The predominant feature of human CSVD involves demyelination, while the microbleed formation accounts for smaller proportion of the findings (3) which is opposite to our findings of CSVD in SHRSP. Taken together, these findings suggest that histopathological changes of the small and medium vessels are similar between human with CSVD and SHRSP. However, the subsequent histological phenotypes of the brain tissue alteration may vary. As previously reported, these differences are inherent to differences in the brain anatomy, white-gray matter ratio, and cerebral perfusion pattern between the humans and rats (4). This further highlights the role of quantitative neuroimaging as an additional tool for detecting the changes associated with CSVD in SHRSP.

Sensorimotor testing identified significant differences in behavior suggestive of motor restlessness associated with hyperactivity during testing in SHRSP compared to WKY at the beginning of the experiment. This is in line with a previous study suggesting SHRSP as a potential model for anxiety and attention deficit hyperactivity disorder while WKY may serve as a potential model for depression (48, 49). These behavioral abnormalities in SHR have been attributed to deficiency in the dopaminergic system while the administration of psychostimulants, such as d-amphetamine alleviated these hyperactivity symptoms (50). Subsequently throughout the experiment, SHRSP has shown a larger decrease in sensorimotor measures compared to WKY reaching a nonsignificant difference level toward the end of the experiment. While the large initial decrease in the activities of both SHRSP and WKY was likely due to habituation to the test, it is possible that the progression of CSVD lesions in SHRSP throughout age has resulted in further worsening of these metrics in SHRSP. These results have significant implications when utilizing SHRSP and WKY as a CSVD model and its control as the underlying behavioral differences between WKY and SHRSP may impact the interpretation of cognitive testing or decline resulting from accumulation of CSVD lesions in this model.

Our study has several limitations that needs to be investigated in future studies. First, future work should assess female SHRSP to understand their CSVD phenotypes and how they may differ from the male ones. In addition, as our study contained a small number of brain MRIs at young age, it is critical to expand the neuroimaging studies of SHRSP to earlier in life to understand the early changes that may be associated with CSVD in particular in brain volumetric measurements. This additional neuroimaging testing will also allow for volumetric assessment of several additional brain regions and adequate correction for multiple comparisons among them. Furthermore, as we focused on concomitant radiographic and histopathological studies at each time point, serial neuroimaging studies are needed to assess the degree, progression and timing of brain, and subregional atrophy in SHRSP. Finally, further studies with concomitant registration of the histological slides along with the MRI sequences will be needed to identify the exact neuroimaging-histopathologic correlates of each identified lesion type.

In conclusion, our study suggests that SHRSP fed with regular diet is a relevant model for hypertension-induced human CSVD. These rats exhibit age-dependent histopathological progression of CSVD lesions. In correlation, brain MRI identifies subcortical T2w hyperintensities with significant differences in the brain and white matter volumes compared to WKY. These findings suggest the utility of brain MRI and quantitative neuroimaging to identify *in vivo* correlates of the disease that can potentially be used as endpoints in future therapeutic studies.

## Data Availability Statement

The raw data supporting the conclusions of this article will be made available by the authors upon request, without undue reservation.

## Ethics Statement

The animal study was reviewed and approved by the Intuitional Animal Care and Use Committee (IACUC) at The Ohio State University and they were conducted in compliance with the Public Health Service Policy on Humane Care and Use of Laboratory Animals.

## Author Contributions

YH designed the research, wrote the manuscript first draft, and secured the study funding. YH, EC, and AB performed the research. YH, AB, KP, JS, CR, ME, and JZ analyzed the data. YH, EC, ME, AB, KP, JS, CR, and JZ revised the manuscript for its intellectual content. All authors contributed to the article and approved the submitted version.

## Funding

This study was funded by the following grants to YH: the Davis Bremer Pre-K Career Development Award from the Center of Clinical Translational Sciences at the Ohio State University, Neurological Research Institute at the Ohio State University, NINDS NS086484 through StrokeNet Research Fellowship to YH, and the William T. Tozer Hemorrhagic Stroke Research Fund.

JS was supported by the National Institute of Disability, Independent Living and Rehabilitation Research (NIDILRR Grant 90SI5020), the National Institutes of Neurological Disorders-NIH (Grant R01 NS118200), the European Union (EU Era Net – Neuron Program, SILENCE Grant 01EW170A), the Craig H Neilsen Foundation (Grant 596764), the Wings for Life Spinal Cord Research Foundation and the William E. Hunt and Charlotte M. Curtis endowment. JS is a Discovery Theme Initiative Scholar (Chronic Brain Injury) of the Ohio State University.

JZ was supported by the NHLBI through the following grants: HL131941 and HL135648. Small Animal Imaging Core at The Ohio State University, where animal MRI was performed, is funded by National Cancer Institute (NCI): P30CA016058.

## Conflict of Interest

The authors declare that the research was conducted in the absence of any commercial or financial relationships that could be construed as a potential conflict of interest.

## Publisher's Note

All claims expressed in this article are solely those of the authors and do not necessarily represent those of their affiliated organizations, or those of the publisher, the editors and the reviewers. Any product that may be evaluated in this article, or claim that may be made by its manufacturer, is not guaranteed or endorsed by the publisher.
