# Supplementary material for: Characterizing the Neuroimaging and Histopathological Correlates of Cerebral Small Vessel Disease in Spontaneously Hypertensive Stroke-Prone Rats
Source: Front Neurol. 2021 Nov 30;12:740298. doi: 10.3389/fneur.2021.740298 (PMC8669961; doi:10.3389/fneur.2021.740298)
Supplement: Supplementary file 1 [file Table_1.DOCX]

**Supplementary table 1: Systolic blood pressure of spontaneously hypertensive stroke-prone rats (SHRSP) and Wistar-Kyoto rats (WKY).**

|  | **WKY** | **SHRSP** |  |
| --- | --- | --- | --- |
| **Age (weeks)** | **Mean (Standard Deviation)** | **Mean (Standard Deviation)** | **P Value** |
| **Systolic Blood Pressure (mmHg)** | | | |
| 7 | 117.8 (19.7) | 128.5 (9.4) | 0.035^*^ |
| 10 | 135.8 (19) | 151.5 (17) | 0.0038^*^ |
| 16 | 141.9 (19.8) | 163.7 (17.9) | 0.0038^*^ |
| 24 | 148 (23) | 182 (6.2) | 0.0011^*^ |
| 32 | 139 (28.8) | 173 (14.7) | 0.0163^*^ |

^*^Statistically significant (P<0.05).

**Supplementary table 2: Sensorimotor testing characteristics of spontaneously hypertensive stroke-prone rats (SHRSP) and Wistar-Kyoto rats (WKY).**

|  | **WKY** | **SHRSP** |  |
| --- | --- | --- | --- |
| **Age (weeks)** | **Mean (Standard Deviation)** | **Mean (Standard Deviation)** | **P Value** |
| **Total Distance Travelled (meter)** | | | |
| 6 | 5.9 (4.6) | 17.1 (4) | <0.0001^*^ |
| 7 | 3.6 (3.7) | 11.2 (5.1) | <0.0001^*^ |
| 8 | 2.2 (1.7) | 8.2 (5.7) | <0.0001^*^ |
| 16 | 1.3 (1.8) | 6.2 (4.4) | <0.0001^*^ |
| 24 | 1.3 (1.2) | 6.9 (7.6) | 0.076 |
| 32 | 1.5 (1.9) | 6.2 (7.5) | 0.12 |
| **Average Speed (meter/second)** | | | |
| 6 | 0.02 (0.016) | 0.057 (0.013) | <0.0001^*^ |
| 7 | 0.012 (0.012) | 0.038 (0.016) | <0.0001^*^ |
| 8 | 0.007 (0.006) | 0.027 (0.019) | <0.0001^*^ |
| 16 | 0.004 (0.006) | 0.021 (0.015) | <0.0001^*^ |
| 24 | 0.004 (0.004) | 0.023 (0.025) | 0.076 |
| 32 | 0.005 (0.007) | 0.021 (0.025) | 0.12 |
| **Total time mobile (seconds)** | | | |
| 6 | 114.1 (59.3) | 202 (34.1) | <0.0001^*^ |
| 7 | 63.5 (46.1) | 128.7 (52.6) | <0.0001^*^ |
| 8 | 41.5 (29.9) | 89.9 (55.9) | 0.0002^*^ |
| 16 | 30.1 (43.7) | 70.1 (43.9) | 0.001^*^ |
| 24 | 23.5 (18.4) | 63.1 (54.5) | 0.083 |
| 32 | 19.5 (26) | 49.7 (57) | 0.2 |

^*^Statistically significant (P<0.05).

**Supplementary table 3: Analysis of subcortical hyperintensities on T2 sequences in Wistar Kyoto rats (WKY) and Spontaneously Hypertensive Stroke-Prone Rats (SHRSP).**

| **Number of T2 subcortical hyperintensities per animal** | | | |
| --- | --- | --- | --- |
|  | **WKY** | **SHRSP** | **P value** |
| **Time point** | **Mean (Standard Deviation)** | **Mean (Standard Deviation)** |  |
| Total study | 0.692(1.11) | 2.933(1.44) | 0.0005* |
| 7 weeks | 0 | 0 | N/A |
| 16 Weeks | 0.333(0.516) | 3(1.633) | 0.014* |
| 24 Weeks | 0.5(0.707) | 3.333(0.577) | 0.07 |
| 32 Weeks | 1.5(1.732) | 3.143(1.345) | 0.12 |
| **Animals with subcortical hyperintensities** | | | |
|  | **Percentage (95% Confidence Interval)** | **Percentage (95% Confidence Interval)** |  |
| Total study | 38.5(13.9-68.4) | 0.933(68.1-99.8) | 0.004* |
| 7 weeks | 0 | 0 | N/A |
| 16 weeks | 33.3(4.3-77.7) | 100(39.8-100) | 0.071 |
| 24 weeks | 50(1.3-98.7) | 100(29.2-100) | 0.4 |
| 32 weeks | 50(6.7-93.2) | 100(59-100) | 0.109 |

*statistically significant association (P<0.05).

**Supplementary table 4: Analysis of brain MRI volumetric data and Diffusion Tensor Imaging (DTI) in Wistar-Kyoto rats (WKY) and Spontaneously Hypertensive Stroke-Prone rats (SHRSP).**

| **Intracranial Volume (mm^3^)** | | | |
| --- | --- | --- | --- |
|  | **WKY** | **SHRSP** | **P value** |
| **Time Point** | **Mean (Standard Deviation)** | **Mean (Standard Deviation)** |  |
| Total Cohort | 2226(121.7) | 2059(100.1) | 0.0001^*^ |
| 7 week | 1876.2 | 1752 | N/A |
| 16 week | 2216.6(27.1) | 2039.5(11.1) | 0.011^*^ |
| 24 week | 2236.2(59.3) | 2132.6(71.2) | 0.08 |
| 32 week | 2322.4(58) | 2082(45.1) | 0.008^*^ |
| **White Matter Volume (mm^3^)** | | | |
| Total Cohort | 59.8(8.8) | 54.4(7.1) | 0.045^*^ |
| 7 week | 41.4 | 33 | N/A |
| 16 week | 56.5(3.8) | 53.7(3) | 0.2 |
| 24 week | 60.7(0.43) | 55.5(2.2) | 0.08 |
| 32 week | 68.9(6.5) | 57.4(4.8) | 0.008^*^ |
| **Hippocampal Volume (mm^3^)** | | | |
| Total Cohort | 85.8(6.1) | 81.6(9.1) | 0.12 |
| 7 week | 92.3 | 68.2 | N/A |
| 16 week | 85.1(6.2) | 83.1(7.2) | 0.39 |
| 24 week | 81.7(1) | 76.7(3.7) | 0.08 |
| 32 week | 86.6(6.5) | 84.8(10.4) | 1 |
| **Fractional Anisotropy (FA) – Corpus Callosum** | | | |
| Total cohort | 0.528(0.037) | 0.527(0.052) | 1 |
| 7 week | 0.531 | 0.481 | N/A |
| 16 week | 0.515(0.046) | 0.526(0.072) | 0.83 |
| 24 week | 0.558(0.024) | 0.553(0.056) | 1 |
| 32 week | 0.533(0.026) | 0.52(0.042) | 0.88 |
| **Mean Diffusivity (MD) – Corpus Callosum (mm^2^/s)** | | | |
| Total cohort | 0.928(0.119) | 0.869(0.093) | 0.009^*^ |
| 7 week | 0.913 | 0.915 | N/A |
| 16 week | 0.909(0.172) | 0.905(0.11) | 0.52 |
| 24 week | 0.971(0.0003) | 0.851(0.072) | 0.08 |
| 32 week | 0.943(0.021) | 0.841(0.107) | 0.053 |
| **Fractional Anisotropy (FA) - External Capsule** | | | |
| Total cohort | 0.383(0.029) | 0.38(0.045) | 0.87 |
| 7 week | 0.34 | 0.3 | N/A |
| 16 week | 0.374(0.025) | 0.384(0.061) | 0.83 |
| 24 week | 0.391(0.02) | 0.405(0.032) | 0.56 |
| 32 week | 0.407(0.014) | 0.379(0.028) | 0.88 |
| **Mean Diffusivity (MD) – External Capsule (mm^2^/s)** | | | |
| Total cohort | 0.902(0.028) | 0.848(0.076) | 0.03^*^ |
| 7 week | 0.896 | 0.868 | N/A |
| 16 week | 0.921(0.02) | 0.872(0.081) | 0.29 |
| 24 week | 0.9(0.007) | 0.886(0.085) | 0.56 |
| 32 week | 0.866(0.014) | 0.802(0.014) | 0.18 |

(^*^) Statistically significant (P<0.05).
